# Proceedings of the Medical Association of the State of Alabama, Begun and Held in the City of Mobile, December, 1850

**Published:** 1851-04

**Authors:** 


PROCEEDINGS OF THE MEDICAL ASSOCIATION OF THE STATE OF ALABAMA, BEGUN AND HELD IN THE CITY OF MOBILE, DECEMBER, 1850.

We have read these proceedings with interest, and we are pleased to see the laudable emulation displayed among the members for the elevation of the medical profession.

The appendix to the proceedings contains an able address, by the President of the Association, Dr. Lopez, Dr. C. F. Percivall's Report of Diseases prevailing in Church Hill, and its vicinity, An account of the Medical Botany of Sumter county, Alabama, Report on the Diseases of Mobile, Diseases, etc. of Lowndesboro', and its vicinity, by Dr. Wooten, on the Diseases of Sumter county by Dr. Anderson, Report of a "wound of the heart; penetrating the right ventricle," from which the patient recovered, by Chas. E. Lavender, M. D., on the Diseases of Wetumpka, by Dr. T. W. Mason, notes on Marasmus, pertussis and typhoid fever, by Dr. Reese, Report on the Diseases of the western part of Lowndes county, by Dr. Reese, Clinical facts relative to the administration of cod-liver oil, by Dr. W. H. Anderson. The Valedictory Address was delivered by Dr. Lavender.

The proceedings of this association are highly creditable to the physicians of Alabama, and they cannot fail to be serviceable to the progress of medicine in the South. B.

